# The consequences of including non-additive effects on the genetic evaluation of harvest body weight in Coho salmon (*Oncorhynchus kisutch*)

**DOI:** 10.1186/1297-9686-42-19

**Published:** 2010-06-11

**Authors:** José A Gallardo, Jean P Lhorente, Roberto Neira

**Affiliations:** 1 Laboratorio de Genética Aplicada, Escuela de Ciencias del Mar, Pontificia Universidad Católica de Valparaíso, Avenida Altamirano 1480, Valparaíso, Chile; 2 Aquainnovo S.A., Polpaico 037, Barrio Industrial, Puerto Montt, Chile; 3 Departamento de Producción Animal, Facultad de Ciencias Agronómicas, Universidad de Chile, PO Box 1004, Santiago, Chile

## Abstract

**Background:**

In this study, we used different animal models to estimate genetic and environmental variance components on harvest weight in two populations of *Oncorhynchus kisutch*, forming two classes i.e. odd- and even-year spawners.

**Methods:**

The models used were: additive, with and without inbreeding as a covariable (A + F and A respectively); additive plus common environmental due to full-sib families and inbreeding (A + C + F); additive plus parental dominance and inbreeding (A + D + F); and a full model (A + C + D + F). Genetic parameters and breeding values obtained by different models were compared to evaluate the consequences of including non-additive effects on genetic evaluation.

**Results:**

Including inbreeding as a covariable did not affect the estimation of genetic parameters, but heritability was reduced when dominance or common environmental effects were included. A high heritability for harvest weight was estimated in both populations (even = 0.46 and odd = 0.50) when simple additive models (A + F and A) were used. Heritabilities decreased to 0.21 (even) and 0.37 (odd) when the full model was used (A + C + D + F). In this full model, the magnitude of the dominance variance was 0.19 (even) and 0.06 (odd), while the magnitude of the common environmental effect was lower than 0.01 in both populations. The correlation between breeding values estimated with different models was very high in all cases (i.e. higher than 0.98). However, ranking of the 30 best males and the 100 best females per generation changed when a high dominance variance was estimated, as was the case in one of the two populations (even).

**Conclusions:**

Dominance and common environmental variance may be important components of variance in harvest weight in *O. kisutch*, thus not including them may produce an overestimation of the predicted response; furthermore, genetic evaluation was seen to be partially affected, since the ranking of selected animals changed with the inclusion of non-additive effects in the animal model.

## Background

Several studies have shown that non-additive effects like common environmental and dominance genetic effects can be an important component in the total phenotypic variance of quantitative traits in fish [1-6]. In salmon breeding, common environmental effects may be observed when members of different families are reared in separate tanks until the fish reach a sufficiently large size for individual physical marking. Common environmental variance represents a proportion of the total phenotype variance and ranges from 0 to 0.09 for growth related traits in salmonids [7,1,3,2,5]. In trout, significant full-sib family effects for body weight have been considered as indirect evidence of dominance variance, ranging from 0.01 to 0.17 [8], however, it may be confused with other non-additive effects or a common environmental effect. Dominance genetic variance representing a proportion of the total phenotypic variance has been reported in various species and ranges from 0 to 0.22 for body weight at harvest in rainbow trout [5], from 0.08 to 0.27 for different measurements of body weight in Chinook salmon [2,9], from 0.02 to 0.18 for body weight at harvest in Atlantic salmon [4], and from 0.16 to 0.34 for swim-up stage weight in brown trout [10]. In the context of animal models, Rye and Mao [4] and Pante et al. [5] have shown that fitting non-additive effects, particularly dominance genetic effects, resulted in a remarkable decrease in the heritability estimate, while the residual variance either remained the same or increased slightly. Thus, the predicted genetic response may be biased upwards if dominance genetic variance is not included in animal models. Pante et al. [10] have suggested that if significant dominance genetic variance is found, studies should be undertaken to determine whether re-ranking of breeding values occurs.

The objective of this study was to investigate the magnitude of dominance genetic and common environmental variances, and the consequences of including these effects plus inbreeding on the genetic evaluation of harvest body weight in O. kisutch. Particularly, we are interested in the effects on heritability, genetic response and on ranking of animals selected as parents.

## Methods

### Studied populations and data structure

The study was based ontwo *O. kisutch*. populations from the Genetic Improvement Center (CMG) maintained by the Institute for Fisheries Development (IFOP) and the University of Chile in Coyhaique (XI Region, Chile). These two populations, termed 'even' and 'odd' year classes, were initiated in 1992 and 1993, respectively, from a common base population and managed in a two-year reproductive cycle. Since the program began, both populations were managed as closed populations, maintained by mating approximately one male with three females. The fish spawned from late April to June; the eggs of each full-sib family were incubated separately, and at the eyed egg stage, 120 families were moved to separate tanks for hatching and kept until fish were individually marked using PIT (passive integrated transponder) tags. Rearing families in separate tanks usually produces a common environmental effect that should increase in magnitude as full-sib families are maintained for a long time under these conditions.To prevent high common environmental effects in harvest and confounding effect with dominance, 60-80 fish from 100 families were individually PIT (Passive integrated transponder) tagged in December, seven months after spawning, when the fish averaged about 5-10 g. Then, fish were transferred to estuary water conditions (Ensenada Baja) where each full-sib family was randomly stocked in equal numbers of fish into three rearing sea-cages. Body weight at harvest (harvest weight) was recorded at about 620 days post-spawning, when the fish weight was on average over 2,500 g. Artificial selection for harvest weight and early spawning was applied for four generations as described and analyzed in Neira et al. [11,12] using a simple animal model.

**Figure 1:**
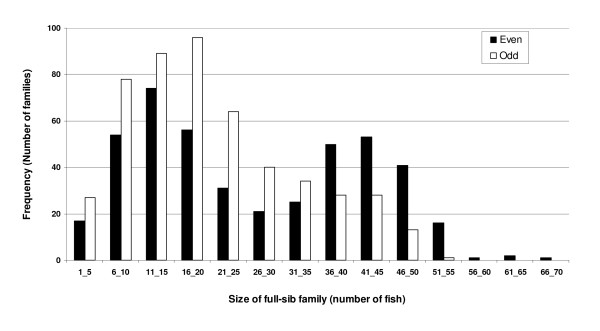
**Frequency distribution of full-sib family sizes in two populations of Coho salmon (even = 442 families; odd = 498 families)**.

The general data structure and the frequency of full-sib family sizes are shown in Table 1 and Figure 1, respectively. The intended design to obtain 100 full-sib families per generation was reached, except for year class 1992 for which only 48 families were formed due to initial infrastructure limitations. A total of 11,833 and 10,327 harvest weight records were analyzed in the even and odd populations, respectively (Table 1), and as expected for a selection experiment, the harvest weight tended to increase with generations. The frequency distribution of full-sib family size at harvest was bimodal for the even population ranging from 2-67 (mean = 26) and unimodal for the odd population ranging from 1-53 (mean = 20). This data structure should allow us to estimate the variance of dominance given that full-sib relationships are present, and because the number of animals per family is adequate.

**Table 1:** Numbers of sires, dams, progeny and average harvest weight standardized to 620 days of age in two populations of *O. kisutch*

Year class	Year	Sires	Dams	Progeny	Harvest weight (g)	Standard deviation (g)
Even	1992	20	48	850	2,667	554
	1994	32	92	937	2,976	495
	1996	27	103	1,796	4,668	823
	1998	30	100	4,458	3,798	990
	2000	34	99	3,792	4,075	1,030
	Total	143	442	11,833	3,873	1,064
						
Odd	1993	36	100	1,229	2,604	410
	1995	32	100	1,177	3,263	497
	1997	33	100	3,864	2,761	542
	1999	30	98	1,901	4,145	785
	2001	43	100	2,156	3,184	535
	Total	174	498	10,327	3,142	779

### Data analysis

The estimation of variance components and the calculation of breeding values were performed with the program AIREMLF90 [13] using single trait animal models as described in Pante et al. [5]. Prior to analysis, the character harvest weight was adjusted to fixed age (620 days), using multiplicative correction factors, to account for the different times of fish growth, so the covariate age was not included in the genetic analysis. Five different animal models were compared, that included the following effects and covariate: random genetic additive effect, with and without inbreeding as a covariate (A + F and A respectively); additive effect plus the random common environmental of full-sibs effect and inbreeding (A + C + F); additive effect plus the random parental genetic dominance effects, and inbreeding (A+ D + F); and a full model (A + C + D + F).

where ***y ***is a vector of observations of animals; ***b ***is a vector of the contemporary group fixed effect year*sea-cage*sex with 30 levels; *a*, *c*, *d *are random effects of additive genetic, common environment due to full-sib families, and dominance respectively; β is the linear regression of ***y ***on inbreeding coefficients; *F *is the coefficient of inbreeding; **X**, **Z**_1_, **Z**_2_, **Z**_3 _are the corresponding incidence matrices relating the effects to ***y***; and ***e ***is the vector of random residuals.

The assumptions for the parameter means (*E*) and variances were:

where  is the additive genetic variance,  is the dominance genetic variance,  is the common environmental variance,  is the error variance, A is the additive genetic relationship matrix, D is the dominance genetic relationship matrix and I is an identity matrix. Note that parental dominance variance is one quarter of the offspring dominance variance [14].

Relative variance components were expressed as ratios of the total phenotypic variance (): heritability (*h*^2^) = ; the dominance variance (*d*^2^) = 4 ; the common environmental variance ratio (*c*^2^) =  for each of the models and for the two populations of *O*. *kisutch*, where  are additive genetic variance, dominance genetic variance and common environmental variance, respectively.

The additive model was compared to other models using likelihood ratio (LR) tests. The likelihood ratio is LR = -2ln[l (θ|y)/l (θr|y)], where l (θ|y) is the maximum of the likelihood function when fitted to a full set of parameters and l (θr|y) is the maximum likelihood, subject to the restriction that r parameters were constrained to fixed values. Asymptotically, the LR test statistic is *χ*^2^_r _distributed, with r degrees of freedom [15].

Genetic responses per generation using the different models were calculated as the difference between mean breeding values in successive generations. To measure the magnitude of a possible over/under estimation of genetic response due to omission of dominance, common environmental effects, and/or inbreeding, the ratio of the genetic response of each model with the simplest model (A) was used.

Performance rankings of animals obtained by different models were compared by: 1) Pearson correlations between estimates of breeding values of the total number of pedigree animals per population and 2) the count of the number of sires and dams that would have been excluded from the selected group using the simplest (A) model (30 best fathers and 100 best dams) in each of the other models.

## Results

Estimation of the variance components and inbreeding coefficient for all models and for the two populations are shown in Table 2. Variance components were different in each population, though the inclusion of non-additive effects and inbreeding produced similar effects on the variance estimates for both populations. Including inbreeding as covariable neither affected the estimation of additive variance and nor significantly increased log likelihood. A high additive variance for harvest weight was estimated in both populations when simple additive models (A + F and A) were used, leading to high heritability estimates for both populations (0.45-0.50) while, additive variance decreased and residual variance increased when dominance or common environmental effects were included. Thus, heritability decreased to 0.21 (even) and 0.37 (odd) when C, D or both were considered in the model. When D was included in the model, the magnitude of dominance variance expressed as a ratio of the total phenotypic variance ranged from 0.19 to 0.21 (even) and from 0.06 to 0.10 (odd). The magnitude of the common environmental effect expressed as a ratio of the total phenotypic variance was lower than 0.01 when D was considered in the model in both populations and was 0.06 (even) and 0.02 (odd) when D was not included. The estimated D and C effects are confounded in both populations because the magnitude of the residual variance increased marginally or remained the same when an effect was added to the model (Table 2).

**Table 2:** Estimates of variance components for harvest weight (standard deviation), inbreeding depression (ID), relative variance components (*h*^*2 *^= heritability; *d*^*2 *^= dominance variance; *c*^*2 *^= common environmental variance ratio), Log likelihood values (-2logL) and likelihood ratio (LR) for the different models for two populations of *O. kisutch*

Population	Model	Additive	Parental dominance	Common environmental	Residual	ID	h2	d2	c2	-2logL	LR
Even	A	357270 (23491)			428280 (12313)		0.45			339004	
	A + F	357590 (23564)			428020 (12346)	-7.5	0.46			339002	-2.8
	A + C + F	147590 (21745)		40144 (5377)	522080 (11624)	-7.4	0.21		0.06	338919	-84.9*
	A + D + F	150160 (21888)	38049 (5181)		520850 (11689)	-6.3	0.21	0.21		338914	-90.5*
	A + C +D + F	148420 (21984)	33649 (16192)	4850 (16531)	521660 (11724)	-6.4	0.21	0.19	0.007	338914	-90.6*
											
Odd	A	184320 (12209)			181320 (6543)		0.50			279858	
	A + F	182410 (12135)			182260 (6515)	-7.2	0.50			279855	-2.5
	A + C + F	131310 (12568)		8094 (1962)	206260 (3706)	-7.4	0.38		0.02	279836	-21.7*
	A + D + F	126370 (12562)	8660 (2040)		208750 (6708)	-7.6	0.37	0.10		279836	-21.8*
	A + C +D + F	127450 (12602)	5470 (8840)	3073 (8604)	208190 (6727)	-7.5	037	0.06	0.009	279836	-21.9*

* indicates a significant difference in minima at P < 0.05 based on the likelihood ratio (LR). LR is the difference between -2log L for the indicated model and Model A, with a negative value indicating improvement in the minimum of the likelihood

As Table 3 shows, the average genetic selection response in harvest weight was 21-22% higher in the even population than in the odd population only when simple models (A and A +F) were used but, when the model included C, D or both, estimation of the genetic response was slightly better in the odd population, between 5 to 10% higher, than in the even population. Analysis of the ratio of the genetic response between models (Table 3) shows that the estimated response is practically the same between both additive models (A and A + F), but using these models overestimated the genetic response between 22-25% in the odd population and 40-41% in the even population.

**Table 3:** Comparison of genetic response and genetic response ratio for harvest weight (g) from different models in two populations of *O. kisutch*

		Genetic response by models				
		
Population	Generation	A	A + F	A + C + F	A + D + F	A + C +D + F
Even	1	351.3	357.8	203.9	218.9	215.9
	2	211.1	213.9	157.4	160.6	159.7
	3	413.3	415.1	246.3	241.1	240.2
	4	521.0	516.8	272.1	274.9	272.7
	Mean	374.2	375.9	219.9	223.9	222.1
						
Odd	1	317.1	314.9	255.7	250.0	251.0
	2	218.6	216.9	175.1	170.2	171.4
	3	430.7	424.2	326.1	316.3	318.6
	4	274.7	271.7	208.7	200.3	202.5
	Mean	310.3	306.9	241.4	234.2	235.9
						
		**Genetic response ratio**				
		
			**A + F/A**	**A + C+ F/A**	**A + D+ F/A**	**A + C + D+ F/A**

Even	1		1.02	0.58	0.62	0.61
	2		1.01	0.75	0.76	0.76
	3		1.00	0.60	0.58	0.58
	4		0.99	0.52	0.53	0.52
	Mean		1.00	0.59	0.60	0.59
						
	1		0.99	0.81	0.79	0.79
Odd	2		0.99	0.80	0.78	0.78
	3		0.98	0.76	0.73	0.74
	4		0.99	0.76	0.73	0.74
	Mean		0.99	0.78	0.75	0.76

Correlations between breeding values estimated with different models were near unity (0.98 to 1.00) suggesting that the breeding values of the selection candidates estimated by the different models do not re-rank (Table 4). However, minor changes observed in the breeding values resulted in some candidate fish for selection obtained in one model to be excluded in another (Table 5). Major differences in selected candidates were observed between both additive models (A and A + F) as compared to models involving dominance effects, common environment effects and both simultaneously (Additional file 1, Table S1 and Table S2). Also, more differences were observed in the even population than in the odd population, which showed the highest dominance variance (Table 5). Small differences of 1-10 excluded candidates (sires and dam) were found between both additive models (A and A + F) and between the models including dominance effects, common environment effects, or both simultaneously.

**Table 4:** Correlation between estimated breeding values with the different models for all animals in the even and odd populations

Population		*A*	A + F	A + C + F	A + D + F	A + C +D + F
Even	A	1.000				
	A + F	1.000	1.000			
	A + C + F	0.982	0.982	1.000		
	A + D + F	0.982	0.983	0.998	1.000	
	A + EC +D + F	0.981	0.982	0.999	1.000	1.000
						
Odd	A	1.000				
	A + F	1.000	1000			
	A + C + F	0.997	0998	1000		
	A + D + F	0.997	0997	1000	1.000	
	A + EC +D + F	0.997	0998	1000	1.000	1.000

**Table 5:** Number of sires and dams that would have been excluded from the group selected by the simple model (A) per year, 30 best sires and 100 best dams, in each of the models

		**N° of sires excluded**				**N° of dams excluded**			
			
**Population**	**Year**	**A + F**	**A + C + F**	**A + D + F**	**A + C + D + F**	**A + F**	**A + C + F**	**A + D + F**	**A + C +D + F**
	
Even	1992	0	14	13	13	1	13	11	11
	1994	1	5	4	4	6	20	20	20
	1996	1	8	8	8	9	25	23	23
	1998	2	6	6	6	3	28	28	28
	2000	4	15	15	15	2	30	31	30
									
Odd	1993	1	5	5	5	0	5	8	7
	1995	1	1	1	1	1	6	7	6
	1997	1	3	5	4	5	14	13	13
	1999	4	8	7	7	4	5	6	6
	2001	2	5	4	4	7	12	10	11

## Discussion

In the present study, the magnitude of additive and non-additive effects was estimated for body weight at harvest in *O*. *kisutch*. In our study, population sizes were almost half (11,000) of that reported by Pante et al. [5] in trout (20,000 individuals per population) and very small to that described by Rye and Mao [4] in Atlantic salmon (50,000-60,000 individuals per population), thus information may not be sufficient to separate with sufficient precision non-additive effects, dominance and full-sib environmental variances [16]. Few studies have addressed this issue in fish, but our results were similar to previous estimates for growth-related traits in other salmonids [2,4,5]. As for the results reported in rainbow trout by Pante et al. [5], including non-additive effects in different models significantly reduced the heritability estimates in both populations studied (even and odd) in comparison with simple models. Consequently, with the reduced heritabilities reported for the models with dominance, the estimates of genetic response per generation reported by Neira et al [12] in both populations appear to be overestimated. These authors have estimated an average genetic response per generation of 383 g (10.5%) and 302 g (9.9%) for the even and odd populations, respectively. These results are very similar to those reported in this study with the simple random models (A, A + F). However, in tour study, we have estimated a genetic response using the dominance model (A + D + F) of 224 and 234 g per generation, which implies overestimations by 40% for the even population and 25% for the odd population. The higher overestimation was produced in the even population for which a higher magnitude of dominance variance was estimated. Differences between even and odd populations have been reported by Gallardo et al. [17], in other areas such as inbreeding rate (even population 2.45% per generation; odd population = 1.10% per generation), and effective population size (even population = 61; odd population = 106).

Including inbreeding coefficients as a covariable did not affect the heritability estimate, which agrees with results previously reported for trout by Pante et al. [5]. In the present study, this may be due to the relatively low level of inbreeding in each population, indeed the inbreeding coefficient after five generations was estimated to reach between 4-10% by Gallardo et al. [17].

Comparison of the rankings of animals between models was performed using two different approximations: a) Correlation of breeding values between models [18,19], and 2) Comparison of the numbers of sires and dams that would have been excluded from the group selected by the simple model per generation, 30 best sires and 100 best dams, in each of the models. High correlations between breeding values suggest that no re-ranking occurs, which agrees with results described by Ferreira et al. [18] who compared full animal models with or without inbreeding in three growth traits in a Hereford cattle population. Changes of ranking, i.e. correlation near 0.5, were observed by Ferreira et al. [18] only when sire models were compared with full models. However, although the correlations between breeding values were high, we observed that some candidates ranking in the top list with the simple models were excluded from the full models. This evidence shows that using simple models do not result only in overestimating genetic response, but also in the possibility that other animals may be selected as breeders.

The results presented in this study show that dominance variance of harvest weight in *O. kisutch*. may be as important as additive variance, in contrast to common environmental effects, which are always small compared to additive and dominance variances. As reported by Pante et al. [5] in trout, we have found evidence that there are confounding effects between dominance and common environments, suggesting that the data structure does not allow us to estimate both components properly.

## Conclusions

Dominance and common environmental variances may be important components of variance of harvest weight in *O. kisutch*, thus not including them may overestimate the predicted response. Genetic evaluation is partially affected, since the ranking of animals is partially changed when including non-additive effects in the animal model. However, the magnitude of these effects may be very different in different populations.

## Competing interests

The authors declare that they have no competing interests.

## Authors' contributions

JAG carried out the data analysis and drafted the manuscript. JPL participated in data capture and helped draft the manuscript. RN conceived the study, participated in its design and coordination and contributed to draft the manuscript. All authors read and approved the final manuscript.

## Supplementary Material

Additional file 1 **Table S1 - Ranking of breeding values estimaed with different models for the Odd population. Sires and dams excluded from the group selected by the simple model per generation, 30 best sires and 100 best dams, in each of the models are marked in bold**. This table shows the ranking based on breeding values obtained for the different models in the odd population. The top sires and dams for each model are colored in each generation for comparative purposes. This allows easy viewing of animals not selected in a model compared to the simplest model. Table S2 - Ranking of breeding values estimaed with different models for the Even population. Sires and dams excluded from the group selected by the simple model per generation, 30 best sires and 100 best dams, in each of the models are marked in bold. This table shows the ranking based on breeding values obtained for the different models in the even population. The top sires and dams for each model are colored in each generation for comparative purposes. This allows easy viewing of animals not selected in a model compared to the simplest model. Click here for file
